# Effect of replacing synthetic nitrogen fertilizer with animal manure on grain yield and nitrogen use efficiency in China: a meta‐analysis

**DOI:** 10.3389/fpls.2023.1153235

**Published:** 2023-05-12

**Authors:** Keyu Ren, Yue Sun, Hongqin Zou, Dejin Li, Changai Lu, Yinghua Duan, Wenju Zhang

**Affiliations:** State Key Laboratory of Efficient Utilization of Arid and Semi-arid Arable Land in Northern China, Institute of Agricultural Resources and Regional Planning, Chinese Academy of Agricultural Sciences, Beijing, China

**Keywords:** manure fertilizer, synthetic N fertilizer, crop yield, nitrogen use efficiency, meta-analysis

## Abstract

To reduce reliance on synthetic nitrogen (N) fertilizer and sustain food production, replacing synthetic N fertilizer with animal manure as an effective method is widely used. However, the effects of replacing synthetic N fertilizer with animal manure on crop yield and nitrogen use efficiency (NUE) remain uncertain under varying fertilization management practices, climate conditions, and soil properties. Here, we performed a meta-analysis of wheat (*Triticum aestivum* L.), maize (*Zea mays* L.), and rice (*Oryza sativa* L.) based on 118 published studies conducted in China. Overall, the results indicated that substituting synthetic N fertilizer with manure increased yield by 3.3%−3.9% for the three grain crops and increased NUE by 6.3%−10.0%. Crop yields and NUE did not significantly increase at a low N application rate (≤120 kg ha^−1^) or high substitution rate (>60%). Yields and NUE values had higher increases for upland crops (wheat and maize) in temperate monsoon climate/temperate continental climate regions with less average annual rainfall (AAR) and lower mean annual temperature (MAT), while rice had higher increases in subtropical monsoon climate regions with more AAR and higher MAT. The effect of manure substitution was better in soil with low organic matter and available phosphorus. Our study shows that the optimal substitution rate was 44% and the total N fertilizer input cannot be less than 161 kg ha^−1^ when substituting synthetic N fertilizer with manure. Moreover, site‐specific conditions should also be considered.

## 1 Introduction

Synthetic nitrogen (N) fertilizer is essential to ensure crop yield and quality in intensive agriculture (Zhang et al., 2013; He et al., 2021). In China, synthetic N fertilizer has been heavily applied in agricultural systems for decades in order to achieve a higher yield per unit area to feed 18% of the global population with only 9% of the world’s arable land (Ren et al., 2022). Even though crop yield has been greatly increased by applying synthetic fertilizer, there is growing evidence that overuse of synthetic N fertilizer has led to large N losses, low N use efficiency (NUE), and substantial environmental risks (e.g., freshwater eutrophication, greenhouse gas emission, and soil acidification) (Guo et al., 2010; Feng et al., 2022; Xu et al., 2022). An overall N surplus of 2.76 × 10^−3^ billion tons (226.9 kg N ha^−1^) was reported in Chinese croplands (Gu et al., 2017), and crop NUE ranges from 25% to 36% (Ju et al., 2009), which is much lower than that in most developed countries (52%–68%) (Yan et al., 2022). Therefore, management practices to reduce the application of synthetic N fertilizer and alleviate agricultural pollution while ensuring efficient crop production are required.

China, as the largest livestock producer in the world, has abundant livestock manure resources (FAOSTAT, 2018). Zhang et al. (2020) reported that livestock manure N had reached 2.3 × 10^−3^ billion tons in 2010, which is approximately half of its fertilizer N consumption. Regrettably, only 40% of livestock manure N is recycled to cropland (Niu and Ju, 2017). Therefore, replacing synthetic N fertilizer with animal manure in agricultural ecosystems may be an effective management practice to reduce the application of synthetic N fertilizer while maintaining crop yields. Multiple studies have found that manure contains abundant nutrients that are readily absorbed by crops, reducing the reliance on synthetic fertilizer (Wang et al., 2017; Zhang et al., 2020; Hou et al., 2022). Replacing synthetic N fertilizer with manure can increase the input of organic carbon into the soil, which is conducive to stimulating mineral N immobilization, thus reducing N losses from croplands (Xia et al., 2017; Liu et al., 2021). Furthermore, synthetic N fertilizer combined with manure can regulate the synchronization of crop N demand with soil N supply to increase crop yield and NUE (Edmeades, 2003; Chen et al., 2014). However, the effects of manure application on crop yield and NUE can be influenced by management practices, soil properties, and climate conditions (Zavattaro et al., 2017; Du et al., 2020). Thus, the reported effect of substituting fertilizer with manure on crop production is inconsistent in many field studies.

An 8-year field experiment on purple soil in southwest China showed that substituting 50% synthetic N with manure increased maize yield by 13.5% and 12.5% compared with full substitution and only synthetic fertilizer, respectively (Xie et al., 2016). However, Liu et al. (2017) found that rice yield did not significantly increase (or even decreased) at different manure substitution rates when compared with synthetic fertilizer. Analogously, there are inconsistent results in the published papers about the effect of manure substitution on NUE (Zhou, 2012; Gao et al., 2015; Liu et al., 2017). Thus, it is necessary to further understand the effect of replacing synthetic N fertilizer with manure on crop yield and NUE so that manure can be used more effectively. As a formal statistical technique, a meta-analysis can integrate independent findings focused on the same purpose and conduct a comprehensive quantitative evaluation (Ding et al., 2018). A recent meta-analysis found that applying manure fertilizer could effectively improve soil quality and crop yield compared with applying synthetic fertilizer alone, but the same total N input was not considered (Du et al., 2020). The work by Zhang et al. (2020) comprehensively evaluated the advantages and disadvantages of replacing synthetic N fertilizer with manure in upland and paddy fields but did not fully consider the impact of climate and soil factors. A global-scale meta-analysis regarding the effect of manure substitution on crop productivity and reactive N losses was conducted but did not consider crop types, N application rates, or soil properties (Xia et al., 2017). Up to now, reports on climate conditions or key soil properties are rare, and the major factors that influence the effect of replacing N fertilizer with manure on crop yield and NUE have not been identified in published analyses. Therefore, it is still unclear in which regions (climate, soil) or under which management conditions the effect of substituting N fertilizer with manure is better.

In this study, we performed a comprehensive meta-analysis to integrate published data (118 peer‐reviewed papers) from field studies in China on the effect of replacing N fertilizer with manure on grain yield and NUE across three major staple crops (wheat, maize, and rice). The objectives of the study were to 1) determine the effects of substituting N fertilizer with manure on the yields and NUE of three crops, 2) quantitatively analyze the effects of various factors (fertilization management practices, climate conditions, and soil properties), and 3) identify major factors influencing the effect of substituting N fertilizer with manure and analyze the relationships between factors and effect size.

## 2 Materials and methods

### 2.1 Data collection

In this study, we used the database of Web of Science (https://www.webofscience.com/wos/alldb/basic-search), Science Direct (https://www.sciencedirect.com/), or China National Knowledge Infrastructure (http://www.cnki.net/) to search for peer-reviewed literature published until 2018. Some keywords were set to perform the search (i.e., manure, grain yield, crop N uptake, and NUE). Meanwhile, we used the following criteria to screen the literature:

i) Trials on wheat, maize, or rice were conducted in Chinese fields.
ii) The literature must include both a control using only synthetic N fertilizer (in which the application rates of P and K can meet crop growth, NPK) and a treatment using manure to completely or partially replace synthetic N fertilizer (NPKM). The total N input of the NPKM treatment must be consistent with that of the NPK treatment.
iii) Treatments needed to have no less than three replicates within an experimental design.
iv) Crop yield, total aboveground N uptake, or NUE had to be reported. The total aboveground N uptake was used to calculate NUE if it was not reported in the studies (Duan et al., 2014):

(1) $$NUE=\frac{N_{t}-N_{0}}{F_{t}}\times 100\%$$

where *N*_t_ and *N*_0_ are the total aboveground N uptake from treatments with N input and without N input, respectively. *F*_t_ is the total N input.

**Figure 1:**
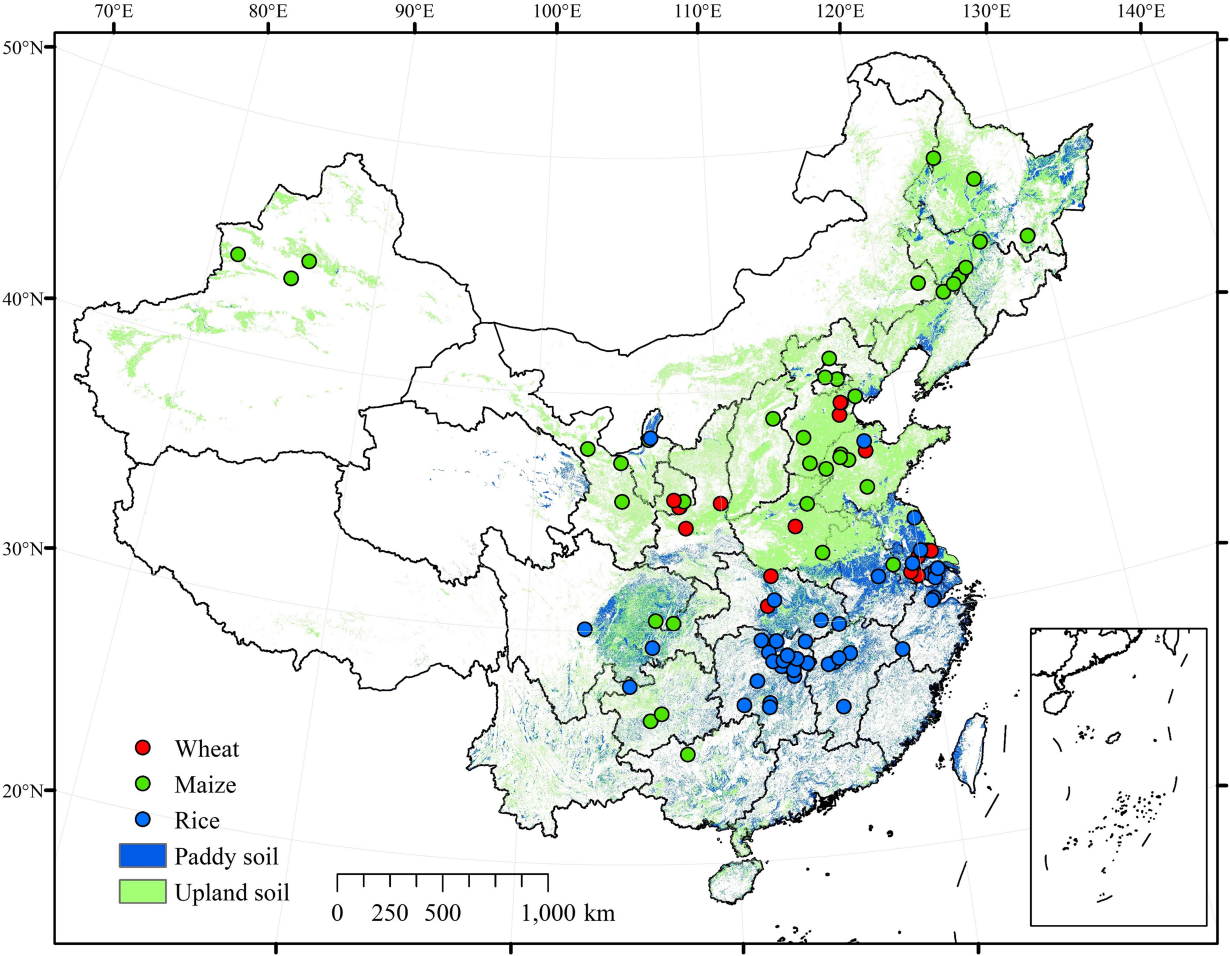
Location of the study sites included in this meta-analysis.

A total of 118 peer-reviewed studies were extracted, including 31 studies on wheat, 53 studies on maize, and 49 studies on rice (**Figure 1**). Some studies included two or more crops. In addition, information on geographical locations, climatic variables [climate type, average annual rainfall (AAR), mean annual temperature (MAT), annual sunshine duration (ASD), and frost-free period (FFP)] for each experiment site, soil properties from a depth of 0 to 20 cm of soil layer [soil organic matter (SOM), soil total nitrogen (TN), soil alkali nitrogen (AN), soil available phosphorus (AP), soil available potassium (AK), and soil pH] in the treatment plots, and the N application rate of manure and synthetic N fertilizer was collected and used for the subgroup analysis.

### 2.2 Evaluated variables and grouping

To evaluate the effects of manure substitution on yield and NUE under different factors, each variable was further divided into subgroups. The N application rates (NR) were grouped into four levels (Yu and Shi, 2015): low (≤120 kg ha^−1^), moderate (120−240 kg ha^−1^), high (240−360 kg ha^−1^), and overuse (>360 kg ha^−1^). There were three substitution rate (SR) groups (Zhang et al., 2020; Ren et al., 2022):<30%, 30%−60%, and >60%. Based on climatic conditions and cropping system, the study areas were divided into three climatic regions (Wang et al., 2013): temperate monsoon climate (NTM), temperate continental climate (NTC), and subtropical monsoon climate (STM). The AAR (Shang et al., 2001), MAT (Fang et al., 2016), ASD (Li et al., 2013), and FFP (Ning et al., 2015) were all grouped into low, moderate, and high levels. The soil nutrient indices were also divided into three levels referring to the “Soil Nutrient Classification Standards” developed by the National Soil Census Office (1998) for a second soil survey in China. Soil pH was categorized into four groups [strongly acidic soils (≤5.5); acidic soils (5.5−6.5); neutral soils (6.5–7.5); alkaline soils (>7.5)]. The specific groups of each variable are shown in **Table 1**.

**Table 1:** Grouping of each variable tested for significance as predictors in yield and NUE response in the meta-analysis.

Variables	Groups
Crop type	Wheat, maize, rice
N application rate (NR, kg N ha^−1^)	≤120, 120−240, 240−360, >360
Substitution rate (SR, %)	≤30, 30−60, >60
Climate type	Temperate monsoon climate (NTM), temperate continental climate (NTC), subtropical monsoon climate (STM)
Average annual rainfall (AAR, mm)	≤600, 600−1,200, >1,200
Mean annual temperature (MAT, °C)	≤12, 12−16, >16
Annual sunshine duration (ASD, h)	≤2,200, 2,200−2,600, >2,600
Frost-free period (FFP, days)	≤175, 175−250, >250
Soil organic matter (SOM, g kg^−1^)	≤20, 20−30, >30
Soil total nitrogen content (TN, g kg^−1^)	≤1, 1−1.5, >1.5
Soil alkali nitrogen content (AN, mg kg^−1^)	≤60, 60−120, >120
Soil available phosphorus content (AP, mg kg^−1^)	≤10, 10−20, >20
Soil available potassium content (AK, mg kg^−1^)	≤175, 175−250, >250
Soil pH	≤5.5, 5.5−6.5, 6.5−7.5, >7.5

### 2.3 Data analysis

In this study, the effect size is used to quantitatively evaluate the effects of replacing synthetic N fertilizer with manure on crop yield and NUE, and it is calculated as the natural logarithm of the response ratio (RR) of NPK and NPKM treatments (Hedges and Curtis, 1999):


(2) $$RR=\ln\frac{{\bar{X}}_{NPK}}{{\bar{X}}_{NPKM}}$$

where ${\bar{X}}_{NPK}$ and ${\bar{X}}_{NPKM}$ represent the mean yield (or NUE) of the NPK and NPKM treatments.

We used the equation $(e^{RR_{++}}-1)\times 100\%$ to calculate the percentage of change in yield (or NUE) from the NPKM treatment compared with the NPK treatment (Ren et al., 2022), where $RR_{++}$ is calculated as follows:


(3) $$RR_{++}=\frac{\sum_{i=1}^{m}\sum_{j=1}^{ki}w_{ij}RR_{ij}}{\sum_{i=1}^{m}\sum_{j=1}^{ki}w_{ij}}$$

where *m* and *ki* represent the number of groups for a given variable (e.g., SR or AAR) and the number of comparisons between the NPKM treatment and the NPK treatment at the *i-*th group, respectively. $RR_{ij}$ and $W_{ij}$ represent the *RR* and weighted factor for the *i*-th group and the *j*-th pair, respectively. $W_{ij}$ is calculated as:


(4) $$w_{ij}=\frac{1}{v}$$

where *v* is a variance:


(5) $$v=\frac{SD_{NPKM}^{2}}{n_{NPKM}{\bar{X}}_{NPKM}^{2}}+\frac{SD_{NPK}^{2}}{n_{NPK}{\bar{X}}_{NPK}^{2}}$$

where $SD_{NPKM}$ and $n_{NPKM}$ represent the standard deviation and the number of samples in the NPKM treatment, respectively, and $SD_{NPK}$ and $n_{NPK}$ represent the standard deviation and the number of samples in the NPK treatment, respectively.

The 95% confidence interval (CI) of $RR_{++}$ is used to evaluate whether the effects of replacing synthetic N fertilizer with manure on crop yield (or NUE) are significant, and it was calculated as follows:


(6) $$\text{CI}=RR_{++}\pm 1.96S\left(RR_{++}\right)$$

(7) $$S\left(RR_{++}\right)=\sqrt{\frac{1}{\sum_{i=1}^{m}\sum_{j=1}^{ki}w_{ij}}}$$

where $S\left(RR_{++}\right)$ represents the standard error of $RR_{++}$ .

In this study, if the 95% CI for yield (or NUE) overlaps with zero, we consider that manure substitution has no significant effect on yield (or NUE) compared with the NPK treatment (*P* > 0.05). Conversely, we consider that it has a significant effect (*P*< 0.05). The meta-analysis was performed by using the MetaWin 2.1 software (Rosenberg et al., 2000).

To explore the major factors affecting the effect of manure substitution, the random forest (RF) algorithm was used to investigate the importance of each variable to effect size. A higher mean squared error for percentage increase [MSE increase (%)] means a more important variable. The RF algorithm was run using the “RandomForest” package in R (version 3.3.3). In addition, we used linear and non-linear regression analyses (at *P*< 0.01) to assess the influence of the top three factors of MSE increase (%) on the effect sizes of yield and NUE (Hou et al., 2022). Microsoft Office Excel 2019 and ArcGIS 10.6 were used for data processing, and SPSS 21.0 was used for statistical analysis.

## 3 Results

### 3.1 Overall effects of substituting fertilizer with manure

**Figure 2:**
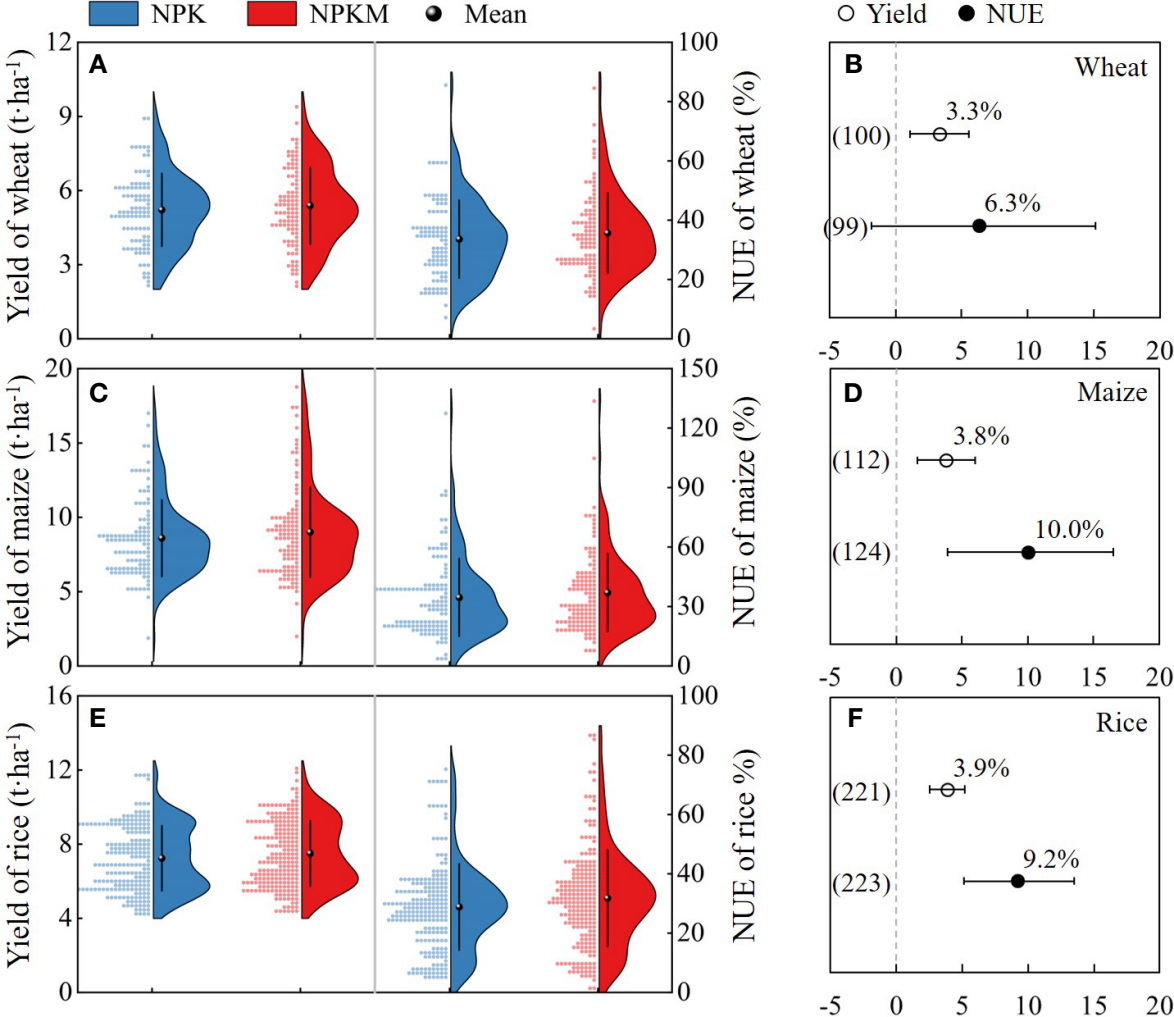
Yield and nitrogen use efficiency (NUE) of wheat **(A, B)**, maize **(C, D)**, and rice **(E, F)** in NPK and NPKM treatments. In **(A, C, E)**, curves are kernel-smoothed distributions fitted to frequency data; balls with error bars indicate mean and standard deviation. In **(B, D, F)**, dots with error bars indicate the overall mean response ratio and 95% CI, and numbers in parentheses represent the independent sample size.

Overall, the average yields were 5.21, 8.59, and 7.23 t ha^−1^ for wheat, maize, and rice, respectively, under the synthetic N fertilizer only (NPK) treatment, and the average NUE values were 33.6%, 34.5%, and 28.8% (**Figures 2A, C, E**). After the substitution of fertilizer N with manure (NPKM), the average respective yields were 5.38, 9.00, and 7.50 t ha^−1^ for wheat, maize, and rice, and the average NUE values were 35.6%, 36.9%, and 31.7% (**Figures 2A, C, E**). The meta-analysis results showed that the NPKM treatment significantly increased yields by 3.3%, 3.8%, and 3.9% for wheat, maize, and rice, respectively, compared with the NPK treatment and significantly increased NUE by 10.0% and 9.2% for maize and rice (**Figures 2B, D, E**). Although the NUE of wheat increased by 6.3%, there was no significant difference between NPK and NPKM.

### 3.2 Effects of fertilization management

**Figure 3:**
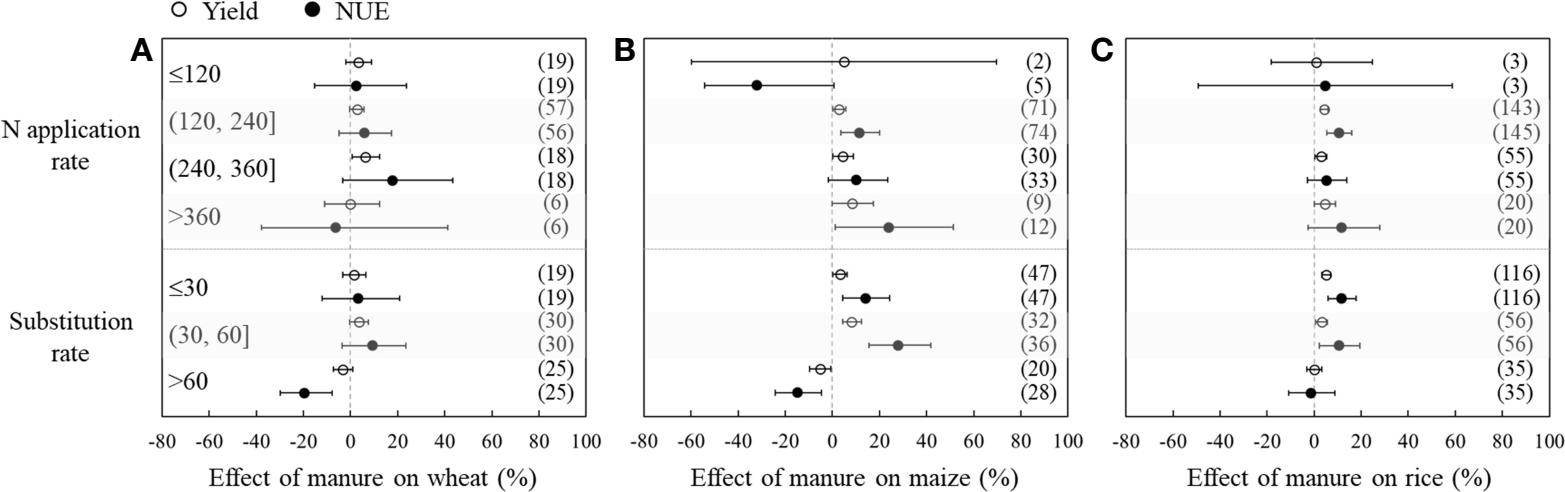
Changes in yield (hollow dots) and nitrogen use efficiency (NUE, black dots) after substitution of synthetic nitrogen fertilizer with manure as affected by fertilization management for wheat **(A)**, maize **(B)**, and rice **(C)**. Dots with error bars indicate the overall mean response ratio and 95% CI, and numbers in parentheses represent the independent sample size.

**Figure 3** depicts how field fertilization management influenced the effects of manure substitution on crop yield and NUE. At low N application rates (≤120 kg ha^−1^), fertilizer substitution with manure did not significantly increase yields or NUE of the three crops. However, the yield significantly increased by 2.7%, 2.9%, and 4.3%, respectively, at moderate N application rates (120−240 kg ha^−1^) for wheat, maize, and rice. The effect of manure on yield increased with increased N application rate (except for wheat with overuse of N fertilizer). The NUE of wheat did not significantly increase at different N application rates, but there were significant increases by 11.5% and 10.6% at moderate N application rates for maize and rice, respectively. For maize, fertilizer substitution with manure significantly increased NUE by 23.8% with the overuse of N fertilizer.

When the rates of substituting manure for fertilizer were less than 60% (SR ≤ 60%), the yield significantly increased by 3.3%−8.2% and 3.1%−5.2% for maize and rice, respectively, and NUE significantly increased by 13.9%−28.0% and 10.5%−11.6%, but there were no significant effects on wheat yield and NUE. However, at a high SR (>60%), the yield decreased by 3.2% and 5.3%, and NUE significantly decreased by 19.7% and 15.0% for wheat and maize, respectively, while there were no significant effects on rice yield and NUE.

### 3.3 Effects of climate

**Figure 4:**
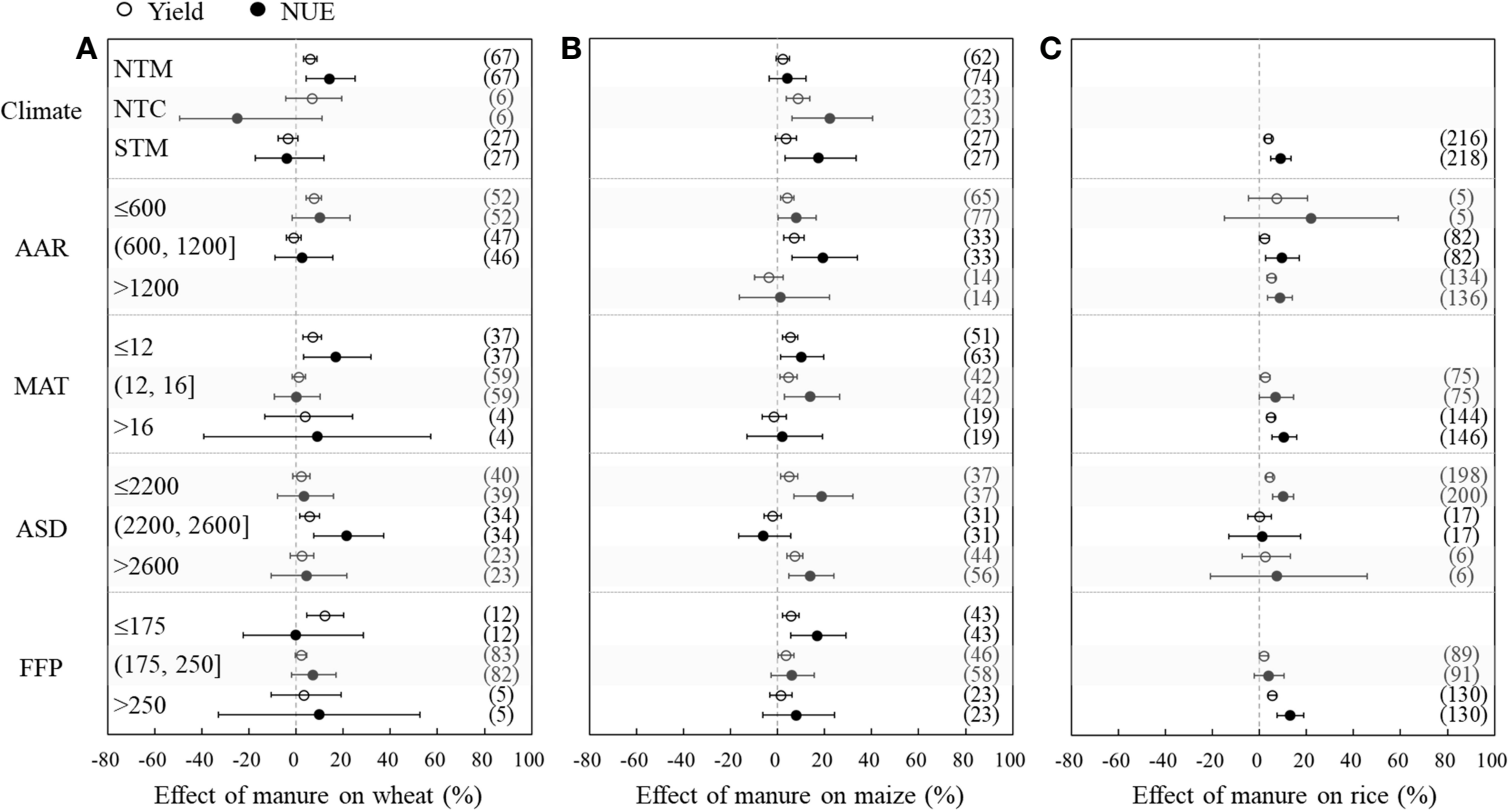
Changes in yield (hollow dots) and nitrogen use efficiency (NUE, black dots) after substitution of synthetic nitrogen fertilizer with manure as affected by climate conditions for wheat **(A)**, maize **(B)**, and rice **(C)**. Dots with error bars indicate the overall mean response ratio and 95% CI, and numbers in parentheses represent independent sample size. NTM, temperate monsoon climate; NTC, temperate continental climate; STM, subtropical monsoon climate; AAR, average annual rainfall; MAT, mean annual temperature; ASD, annual sunshine duration; FFP, frost-free period.

The effects of replacing N fertilizer with manure on crop yields and NUE were different under different climate conditions (**Figure 4**). Yield and NUE significantly increased in the NTM region for wheat, in the NTC region for maize, and in the STM region for rice. Wheat yield and NUE increased by 7.5% and 10.0% by manure application in the regions with AAR ≤600 mm, respectively. In the regions with AAR ≤1,200 mm, maize yield and NUE significantly increased by 4.1%−6.9% and 8.0%−19.2%, respectively. Rice yield and NUE significantly increased in the regions with AAR >600 mm. The yield and NUE were significantly increased in the regions with MAT ≤12°C for wheat and MAT ≤16°C for maize, while rice yield and NUE were significantly increased in the regions with MAT >12°C. The response to manure substitution in wheat, maize, and rice varied in different ASD regions. Wheat yield and NUE significantly increased by 5.7% and 21.3% in the regions with 2200 h< ASD ≤ 2600 h, respectively. In contrast, maize yield and NUE significantly increased in the regions with ASD ≤ 2200 h and 2600 h< ASD. Rice yield and NUE significantly increased by 4.2% and 10.0% in the regions with ASD ≤2200 h. In different FFP regions, manure substitution led to an increase in wheat yield and maize NUE only in the regions with FFP ≤175 days, while the maize yield significantly increased in the regions with FFP ≤250 days. For rice, yield and NUE significantly increased in the regions with FFP >250 days.

### 3.4 Effects of soil properties

**Figure 5:**
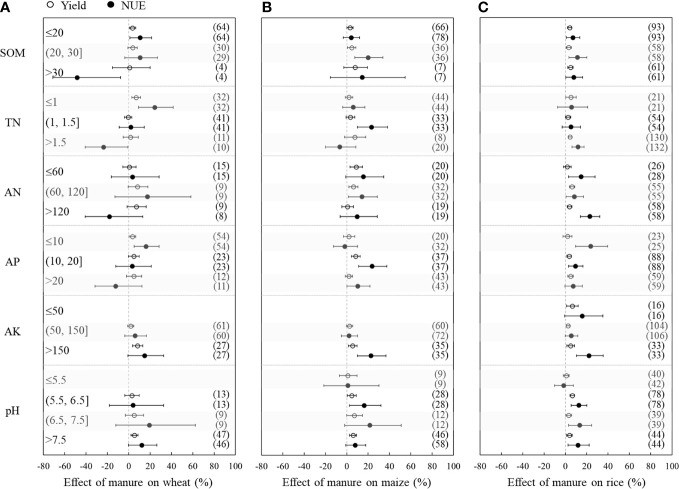
Changes in yield (hollow dots) and nitrogen use efficiency (NUE, black dots) after substitution of synthetic nitrogen fertilizer with manure as affected by soil properties for wheat **(A)**, maize **(B)**, and rice **(C)**. Dots with error bars indicate the overall mean response ratio and 95% CI, and numbers in parentheses represent the independent sample size. SOM, soil organic matter; TN, soil total nitrogen; AN, soil alkali nitrogen; AP, soil available phosphorus; AK, soil available potassium; pH, soil pH.

The responses of crop yields and NUE to manure substitution were affected by soil properties (**Figure 5**). At a low SOM (≤20 g kg^−1^), wheat yield and NUE significantly increased by 3.5% and 10.8%, respectively. However, wheat NUE significantly decreased by 48.5% at SOM >30 g kg^−1^. For maize, yield and NUE significantly increased at 20 g kg^−1^< SOM ≤ 30 g kg^−1^. Rice yield (3.0%−4.8%) and NUE (7.1%−11.4%) significantly increased under different SOM contents. The responses of NUE for the three crops to the substitution of N fertilizer with manure varied for different TN contents. The NUE of wheat significantly increased at TN ≤1 g kg^−1^, while it significantly decreased at TN >1.5 g kg^−1^. The NUE of maize and rice significantly increased only at 1 g kg^−1^< TN ≤ 1.5 g kg^−1^ and TN > 1.5 g kg^−1^, respectively. The yield and NUE of wheat had no significant variations with different AN contents, whereas there were significant increases at 60 mg kg^−1^< AN ≤ 120 mg kg^−1^ for maize and significant increases at AN >60 mg kg^−1^ for rice. At different AP content levels, the NUE values of wheat (16.1%) and rice (23.8%) had the highest increases at AP ≤10 mg kg^−1^, while maize (23.7%) had the highest increase at 10 ≤ AP< 20 mg kg^−1^. In contrast, the yield and NUE of all three crops significantly increased at high AK content (AK > 250 mg kg^−1^). The substitution of fertilizer with manure had no significant influence on crop yields and NUE in strongly acidic soils (pH ≤ 5.5) (there were insufficient data on wheat) but significantly increased crop yields (wheat: 5.3%, maize: 5.8%, rice: 3.8%) in alkaline soils (pH > 7.5). Furthermore, maize yield (4.6%) and NUE (16.3%) significantly increased in acidic soils (5.5< pH ≤ 6.5). For rice, except in strongly acidic soils, substituting manure for fertilizer significantly increased yield and NUE by 3.1%−6.5% and 11.9%−13.3%, respectively.

### 3.5 Main factors influencing the effect of substituting N fertilizer with manure

**Figure 6:**
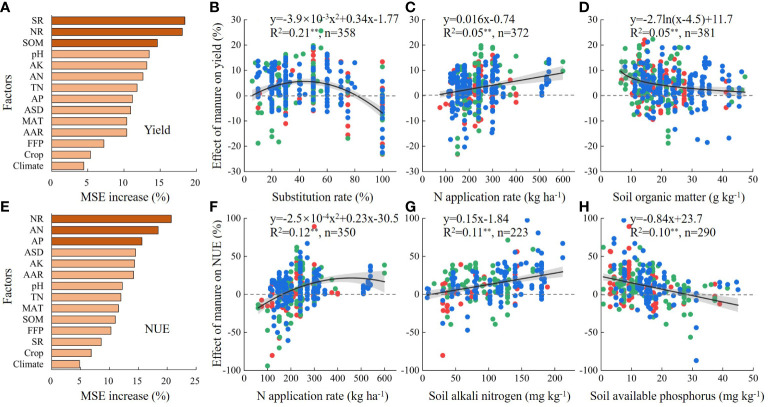
Major factors influencing the effect of substituting synthetic nitrogen fertilizer with manure on yields **(A–D)** and nitrogen use efficiency (NUE) **(E–H)**. The importance of each factor was determined based on mean squared error (MSE) for percentage increase by using random forest models **(A, E)**. Different crop types are indicated by different colors in **(B–D, F–H)**: red = wheat, green = maize, blue = rice. *R* ^2^, coefficient of determination; ** represents significance (*P*< 0.01); *n*, number of observations; NR, N application rate; SR, synthetic N fertilizer substitution rate; SOM, soil organic matter; TN, soil total nitrogen; AN, soil alkali nitrogen; AP, soil available phosphorus; AK, soil available potassium; pH, soil pH; AAR, average annual rainfall; MAT, mean annual temperature; ASD, annual sunshine duration; FFP, frost-free period.

**Figure 6** depicts the importance of variables and correlations between the major factors and the effect size of substituting N fertilizer with manure on yield and NUE. Overall, SR, NR, and SOM were the most important factors for yield. When the substitution rate was 44%, yield had the highest increase (5.6%). The effect of manure on yield was positively correlated with NR but negatively correlated with SOM. The importance of variables on NUE was different from those on yield. NR, AN, and AP were the most important factors for NUE. When the NR >161 kg ha^−1^, the manure substitution had a positive effect on NUE. Furthermore, the effect of manure on NUE increased with the increase of AN, while it decreased with the increase of AP.

## 4 Discussion

### 4.1 Responses of crop yield and NUE to N fertilizer substitution with manure

Overall, our meta-analysis indicates that replacing synthetic N fertilizer with manure increased crop yields and NUE by 3.3%−3.9% and 6.3%−9.2%, respectively (**Figure 2**). Xia et al. (2017) showed that manure substitution increased yields and NUE of grain crops by 5.2% and 10.4%, respectively, based on a global-scale meta-analysis. This suggests that the effect of manure substitution on crop production in China is basically consistent with that in other countries. The application of manure can increase the synchronization of nutrient supply, improve soil properties, and provide extra nutrients. First, the application of manure can increase the microbial immobilization for N owing to the organic carbon supply and then release more evenly throughout the crop growing season (Choi et al., 2001; Ren et al., 2019). Second, the input of manure fertilizer can improve soil aggregate structure, soil water-holding capacity, and microbial activity, thus providing a more favorable soil environment for crop growth (Liu et al., 2010; Zhang et al., 2016). Third, manure fertilizer also provides additional essential micronutrients and nutrients (e.g., copper, zinc fatty acids, proteins, and polysaccharides), which also facilitate the improvement of crop yield and NUE (Hou et al., 2022).

However, crop yields and NUE decreased (or showed no significant effect for rice) when the substitution rate was more than 60% (**Figure 3**), and these results are consistent with recent studies (Xia et al., 2017; Zhang et al., 2020). This likely occurred because the high manure substitution rate does not provide sufficient N for early crop growth because manure N needs a longer time to mineralize than synthetic N fertilizer (Zhou et al., 2016). Similarly, crop yields and NUE did not significantly increase (and even decreased for maize NUE) at N application rates less than 120 kg ha^−1^ (**Figure 3**), which is also due to the insufficient and untimely supply of synthetic N for crop growth following substitution of synthetic N fertilizer with manure. Therefore, it is extremely important to ensure the optimal substitution rate by balancing the application rate of manure and synthetic N fertilizer.

## 4.2 Effects of climate and soil factors on manure substitution

This study also explored the impact of climate conditions and soil properties on the effects of manure substitution (**Figures 4**, **5**). From the perspective of climate factors, relatively higher increases for wheat and maize were observed in the NTM/NTC climate regions with less AAR and lower MAT (**Figures 4A, B**). This is likely because manure was able to exert its heat preservation and water retention properties and stimulate the activity of microorganisms in the soil, to promote the absorption of nutrients by crops in these regions (Kallenbach and Grandy, 2011; Ma, 2018). In addition, the autotrophic nitrification rate is higher in arid climates, and organic N is more easily transformed into ${\text{NO}}_{3}^{-}-\text{N}$ , which wheat and maize prefer to absorb (Zhang et al., 2011; Zhang et al., 2018). Previous studies also showed that organic fertilizer significantly improves wheat yield and NUE in drought years (Ma, 2018). However, a relatively higher increase for rice was observed in the STM climate regions with more AAR and higher MAT (**Figure 4C**). In these regions, synthetic N was lost more easily, while substituting synthetic N fertilizer with manure can slowly release nutrients required by rice (Wang et al., 2015). Moreover, organic N is more easily transformed into ${\text{NH}}_{4}^{+}-\text{N}$ , which rice prefers to absorb in humid climate conditions (Cheng et al., 2019).

In general, the positive effects of substituting synthetic N fertilizer with manure on yield and NUE were relatively high in low soil nutrient conditions (SOM, TN, and AP, **Figure 5**) because the crop yields and NUE were already high with only synthetic N fertilizer in high fertility soil, while the space for further improvement is lower. In contrast, the C source and other nutrients are provided by manure in a more timely and more effective way in low-fertility soil, which can improve soil fertility and promote nutrient absorption in crops (Du et al., 2020). However, yield and NUE did not significantly increase in low AN soil, which may have the same explanation as for the N fertilizer application rate (Zhou et al., 2016). Crop yield and NUE significantly increased in high AK soil, because manure application can alleviate the competitive effect of K^+^ and NH_4_^+^ when crops absorb nutrients (Ren et al., 2019). Furthermore, the crop yields and NUE had no significant difference on the soil at pH ≤5.5 in this study, which may indicate that applying manure fertilizer on strongly acidic soil cannot completely improve crop production and that lime and other soil amendments are also needed (Guo et al., 2010).

### 4.3 Driving factors and recommendations for substituting N fertilizer with manure in grain crops

The results of the random forest model analysis showed that fertilization management (N application and substitution rate) and soil factors (SOM, AN, and AP) were the main factors influencing the effect of manure substitution (**Figure 6**), which is basically consistent with a recent study in paddy fields reported by Hou et al. (2022). Overall, our meta-analysis indicates that substituting synthetic N fertilizer with manure has great potential for improving crop production in China. However, manure substitution in field production needs site-specific strategies to effectively address the impacts of climate factors and soil properties on crop yield and NUE. The reasonable range of substitution rates was 6%−82% and the optimal substitution rate was 44%. The total N fertilizer input cannot be less than 161 kg ha^−1^ when substituting synthetic N fertilizer with manure. From the perspective of crop yields and NUE, the measures of substituting fertilizer with manure can achieve better effects in regions with low SOM and AP than in regions with high AN.

### 4.4 Limitations and uncertainties of the analysis

In this study, the effects of fertilization management practices (NR, SR), climate conditions, and soil properties on yield and NUE of three grain crops (wheat, maize, and rice) were analyzed independently. In fact, they might have interactions, but these were not analyzed due to the lack of data. The effect of manure substitution on yield and NUE is different under different experimental durations because continuous manure application will improve soil structure and fertility over time (Duan et al., 2014). However, experimental durations were not considered in our work, which may cause some uncertainties. In addition, the influencing factors of yield and NUE may be different between wheat, maize, and rice, and each crop needs to be evaluated separately.

## 5 Conclusion

In this study, we performed a meta-analysis to determine the effects of replacing synthetic N fertilizer with manure on crop yield and NUE in China. Substituting synthetic N fertilizer with manure significantly increased yield by 3.3%−3.9% for three grain crops and increased NUE by 6.3%−10.0%. The effect of manure substitution varied under different fertilization management practices, climate conditions, and soil properties. The optimal substitution rate was 44% in practice, and the total N fertilizer input cannot be less than 161 kg ha^−1^ when substituting synthetic N fertilizer with manure. In regions with low soil fertility, the strategy of substituting fertilizer with manure should be considered to improve crop production.

## Data availability statement

The original contributions presented in the study are included in the article/supplementary material. Further inquiries can be directed to the corresponding authors.

## Author contributions

YD designed the research. YS, HZ, and DL provided help with data collection. KR analyzed the data and wrote the manuscript. YD, WZ, and CL revised the manuscript. All authors contributed to the article and approved the submitted version.
